# Changes in resource insecurity, sexual and mental health among young women after Kenya’s 2024 heavy rains and floods

**DOI:** 10.1093/inthealth/ihag064

**Published:** 2026-06-24

**Authors:** Carmen H Logie, Zerihun Admassu, Aryssa Hasham, Julia Kagunda, Humphres Evelia, Rachel Leggett, Clara Gachoki, Mumbi Mwangi, Lesley Gittings, Caetano Dorea, Janet M Turan, Lawrence Mbuagbaw

**Affiliations:** Factor-Inwentash Faculty of Social Work, University of Toronto, 246 Bloor St W, Toronto, ON M5S 1V4, Canada; United Nations University Institute for Water, Environment, and Health, 225 East Beaver Creek Road, Richmond Hill, ON L4B 3P4, Canada; Women’s College Research Institute, Women’s College Hospital, 76 Grenville St, Toronto, ON M5S 1B2, Canada; Factor-Inwentash Faculty of Social Work, University of Toronto, 246 Bloor St W, Toronto, ON M5S 1V4, Canada; Factor-Inwentash Faculty of Social Work, University of Toronto, 246 Bloor St W, Toronto, ON M5S 1V4, Canada; Elim Trust, Jacaranda Drive 71, Mushroom Gardens, Kiambu, Kenya; Centre for the Study of Adolescence, Mbaazi Avenue off Kingara Road, Nairobi, Kenya; Factor-Inwentash Faculty of Social Work, University of Toronto, 246 Bloor St W, Toronto, ON M5S 1V4, Canada; Elim Trust, Jacaranda Drive 71, Mushroom Gardens, Kiambu, Kenya; Elim Trust, Jacaranda Drive 71, Mushroom Gardens, Kiambu, Kenya; School of Health Studies, Faculty of Health Sciences, Western University, 151 Richmond St, London, ON N6A 5B9, Canada; Centre for Social Science Research, University of Cape Town, Cape Town, South Africa; Department of Civil Engineering, University of Victoria, Victoria, BC V8W 2Y2, Canada; Department of Health Policy and Organization, University of Alabama at Birmingham, 1665 University Boulevard, Birmingham, AL 35294, United States; Department of Health Research Methods, Evidence and Impact, McMaster University, Hamilton, ON L8S 4L8, Canada; Department of Anesthesia, McMaster University, Hamilton, ON L8S 4L8, Canada; Department of Pediatrics, McMaster University, Hamilton, ON L8S 4L8, Canada; Biostatistics Unit, Father Sean O'Sullivan Research Centre, St Joseph’s Healthcare, Hamilton, ON L8N 3K7, Canada; Centre for Development of Best Practices in Health (CDBPH), Yaoundé Central Hospital, Yaoundé, Cameroon; Division of Epidemiology and Biostatistics, Department of Global Health, Stellenbosch University, Cape Town, South Africa

**Keywords:** flood, Kenya, mental health, resource insecurity, sexual health, young women

## Abstract

**Objectives:**

Kenya’s 2024 heavy rains and flooding resulted in agricultural devastation and displacement. We assessed resource insecurity, sexual health, and mental health differences across two time points, during this heavy rain/flooding and at 6-month follow-up under dry conditions, among adolescent girls and young women (AGYW) in Kenya.

**Methods:**

We conducted surveys at Time 1 (T1) (heavy rains/flooding, June 2024) and Time 2 (T2) (dry conditions, November 2024) with a non-random sample of AGYW aged 16–24 years in Nairobi and Kisumu. We conducted longitudinal analyses using generalized estimating equations to assess differences over time in resource insecurities (food, water, sanitation, menstruation), sexual health (transactional sex, sexual relationship power (SRP), condom use self-efficacy (CUSE)), and mental health (depression, eco-anxiety), adjusting for sociodemographics and baseline scores, and tested motherhood and traumatic exposure severity as moderators.

**Results:**

Among 586 participants (mean age 20.13 (SD 2.5) years) at follow-up (98.2% retention) vs baseline across sites we found significant resource insecurity reductions (food, sanitation), sexual health improvements (reduced transactional sex, increased SRP), and reduced eco-anxiety. Depression and CUSE changes varied across sites.

**Conclusion:**

Participants reported differences over time in resource insecurity and sexual and mental health. The findings signal the importance of weather-informed health research with AGYW in Nairobi and Kisumu.

## Introduction

Climate change is contributing to increases in the intensity, frequency, and duration of flooding events in Kenya, posing significant risks to health and well-being.^1^ Heavy rains in Kenya from 1 March to 18 June 2024 resulted in 315 fatalities and displaced ∼293 000 people, in addition to adversely impacting health facilities and destroying roads, farmlands, and water sources.^2^ Flooding has serious health impacts, including increases in water-borne, vector-borne, and zoonotic diseases, and physical injury.^3,4^ Flooding also contributes to resource insecurities—including food, water, and sanitation—which in turn can impact physical, mental, and sexual health through complex pathways.^3,5^ Little is known of the experiences of adolescent girls and young women (AGYW) of resource insecurity and health following Kenya’s 2024 flooding and heavy rains. This is an important area to explore, as the effects of extreme weather events (EWEs) may exacerbate gender inequities and gendered health disparities.^5^

Flooding has been associated with worse sexual health outcomes, largely affecting women in diverse sub-Saharan Africa (SSA) countries, including increased HIV prevalence,^6^ elevated gender-based and intimate partner violence (IPV),^6^ reduced access to sexual health resources,^6,7^ and increased sexual practices that elevate HIV exposure, including condomless sex, transactional sex, and multiple sex partners.^8^ These pathways from EWEs such as flooding to worse sexual health outcomes are complex and include infrastructure damage, economic shocks, displacement, and increased household and intrapersonal stressors.^6,9^ Qualitative research with Kenyan young adolescents found that heavy rains and flooding contributed to food and sanitation insecurity, leading to increased sexual violence and transactional sex to meet survival needs (e.g. food), which in turn elevated sexually transmitted infection and HIV acquisition risks and unplanned adolescent pregnancy.^5^ While evidence is growing regarding the associations between climate change-related EWEs and worse sexual health among women in low- and middle-income countries,^6,9^ limited studies have focused specifically on AGYW.

Emerging evidence also demonstrates associations between experiencing an EWE and increased psychological distress and mental health challenges such as depression.^10–12^ Studies have reported direct (e.g. anxiety symptoms) and indirect (e.g. elevated community violence) mental health stressors following EWEs, yet there is a scarcity of longitudinal studies in SSA.^10,11^ This is a notable gap as regional EWEs are increasing globally, and systematic review authors noted that the ‘dearth of studies on the mental health impacts amongst children and adolescents exposed to EWE in SSA is alarming’.^11^

Climate-affected regions and communities in Kenya, as in other SSA settings, which may be particularly vulnerable to the impacts of flooding, include fishing communities and informal settlements.^3,12,13^ However, much EWE-related research on fishing communities focuses on the impacts of flooding on fisherfolk’s livelihoods and potential climate adaptation strategies, leaving knowledge gaps on mental and sexual health among adolescents and youth in these communities.^13^ Research in Kenyan informal settlements suggests these communities are particularly vulnerable to negative impacts of flooding due to inadequate housing, water, and sanitation infrastructure, thus increasing destruction of residents’ homes, sanitation facilities, and resources, and multiplying exposure to health risks.^14^ Studies in informal settlements in SSA at large have suggested that women in these environments may be especially vulnerable to both the health and social impacts of climate-related EWEs including floods, but there is a need for more evidence focused on AGYW.^11,12^ Additional information on flooding-related social and well-being impacts among young women in climate-affected settings could inform gender, age, and contextually tailored climate-informed health programming and research on climate adaptation and risk mitigation.^3,11^

Most research on the impacts of flooding relies on cross-sectional data, partly due to the methodological challenges with collecting longitudinal data before, during, and after a flood.^15^ We aimed to examine longitudinal differences in resource insecurities (food, water, sanitation, menstruation), sexual health, and mental health across two time points spanning periods of heavy rains/flooding and dry conditions among AGYW in Nairobi and Kisumu, Kenya.

## Methods

### Study design and settings

This analysis draws on longitudinal data collected from a cohort study ‘Rada ya Weather’ (Swahili for ‘What’s up with the weather’) at two time points (June 2024, November 2024) with surveys conducted 6 months apart. This study focuses on two climate-affected settings in Kenya, consisting of Nairobi’s informal settlements (Majengo and Mathare) and Kisumu’s rural and peri-urban fishing communities near Ogal and Rota Beaches, which experience EWEs such as drought, flooding, and extreme heat. These two settings are also disproportionately affected by HIV, with an HIV prevalence higher than Kenya’s national average of 4.3% among women (Nairobi: 5.6%, Kisumu: 18.7%).^16^

This community-based study involved a collaboration between academic and community-based Kenyan collaborators, Elim Trust in Nairobi and the Centre for the Study of Adolescence (CSA) in Kisumu. Recruitment used non-random sampling and was conducted by community collaborators and 16 AGYW hired and trained as peer navigators (eight per location). There was a variety of recruitment strategies employed (e.g. coupons, word-of-mouth, community collaborator outreach). The inclusion criteria were AGYW aged 16–24 years; self-reported HIV-negative at baseline; access to a mobile phone; able to communicate in English, Swahili, or Luo; in the past 14 days had experienced one or more resource insecurity (food, water, and/or sanitation) and/or EWE (e.g. drought, flooding, extreme heat); and reported any past 14-day HIV acquisition risk factor (young motherhood, transactional sex, condomless sex, multiple sex partners, IPV). We also intentionally included contextually specific populations of AGYW disproportionately affected by HIV in Kenya, including those involved in Kisumu’s fishing trade^17^ (e.g. young women who buy, sell, and/or clean fish) and young mothers in Nairobi.^18^ Participants who did not meet the inclusion criteria, were not sexually active, or were unable to commit to participation for the study duration, were excluded.

Trained staff from community-based partner organizations administered surveys on tablets using SurveyCTO, a secure platform supporting multiple languages. Tools were piloted with peer navigators and local stakeholders to enhance clarity and ensure cultural relevance. Data collectors underwent protocol and ethics training, and the research team performed daily quality reviews to ensure data accuracy and consistency. Consent materials and surveys were translated into Swahili and Luo. The target sample size was a minimum of 500 to enable longitudinal and site-stratified analyses.^19^

### Measurements

Details of all survey measures, including their recall periods and scoring guidelines, are provided in Table 1. We used longitudinal data from two time points, collected during a period of heavy rains/flooding and 6 months later under dry conditions, to assess changes in resource insecurities. To assess water insecurity we used the Household Water Insecurity Experiences scale (HWISE)].^20^ We categorized water insecurity using the validated HWISE cut-off (<12 vs ≥12) to indicate any experience of water insecurity, ensuring comparability with prior studies and established practice. We used continuous scale measures for: food insecurity: Household Food Insecurity Access Scale (HFIAS);^21^ sanitation insecurity: Caruso *et al*.’s Sanitation Insecurity Scale;^22^ and menstruation insecurity: the Menstrual Insecurity Measure.^23^ We assessed changes in sexual health outcomes including: condom use self-efficacy (CUSE) with the Condom Use Self-Efficacy Scale;^24^ transactional sex; and sexual relationship power (SRP) using the Sexual Relationship Power Scale.^25,26^ We also assessed mental health outcomes: depression, using the nine-item Patient Health Questionnaire (PHQ-9),^27^ and eco-anxiety with the Hogg Eco-Anxiety Scale.^28^ We assessed EWE exposure (cumulative exposure to various types of EWEs and frequency of each exposure) using items from prior research.^29–35^ Covariates included sociodemographic characteristics and the intensity of EWE-related stressors assessed with the Traumatic Exposure Severity Scale (TESS).^36^

**Table 1:** Details of survey measures.

Construct	Description	
Resource insecurity		
Water insecurity	Examined over the past 4 weeks using the 12-item HWISE (Cronbach’s alpha=0.93)	A cut-off point of ≥12 is used to categorize water insecurity
Food insecurity	Measured over the past 4 weeks using the 9-item HFIAS (Cronbach’s alpha=0.94)	Scales for food, sanitation, and menstruation insecurity were each scored by summing the responses to the items, with higher scores indicating greater insecurity on the respective scales
Sanitation insecurity	Assessed in the past 30 days with the 15-item Sanitation Insecurity scale (Cronbach’s alpha=0.97)	
Menstruation insecurity	Assessed during last two menstrual cycles using the 10-item Menstrual Insecurity Measure (Cronbach’s alpha=0.94)	
Sexual health		
CUSE	Evaluated with the 5-item CUSE Scale (Cronbach’s alpha=0.83)	Higher scores indicated greater confidence in condom negotiation and use
Transactional sex	Measured with a single question. At baseline (referring to the past 12 months) and at Time 2 (referring to the past 6 months, since the last survey), participants were asked: ‘Have you had sex (oral, vaginal, or anal) in exchange for any of the following?’ with 11 options (place to stay, food, water, transportation, gifts, school fees, money, drugs, alcohol, clothing, menstrual products)	Those who selected any were classified as transactional sex engaged
SRP	Examined using the 15-item Relationship Control Subscale from the SRP Scale (Cronbach’s alpha=0.89)	Higher scores indicating greater relationship agency
Mental health		
Depression	Assessed depression symptoms over the past 2 weeks with the 9-item PHQ-9 (Cronbach’s alpha=0.89)	Higher scores indicated greater depression severity
Eco-anxiety	Assessed using the Hogg Eco-anxiety Scale, a 10-item scale (Cronbach’s alpha=0.90) that evaluated the frequency of emotional distress related to climate change over the past 2 weeks	Higher scores indicated greater eco-anxiety
Covariates		
Baseline sociodemographics	Age (continuous), education (binary: less than secondary vs secondary or higher), employment (categorical: employed, unemployed, or student), relationship status (categorical: no current partner, married, dating, or casual/multiple partners), parenthood status (binary: yes or no)	Categorized as continuous, binary or categorical (described previously)
Past-year cumulative EWE exposure	Assessed EWE exposure with a range of responses (e.g. drought, flooding, extreme heat). At baseline (Time 1) we asked how many EWEs were experienced in the past 12 months, and at Time 2 we asked how many EWEs were experienced over the past 6 months/since last survey	Summed and categorized into cumulative exposure to multiple types (1, 2–5, >5)
Frequency of past-year EWE exposure	We assessed frequency of exposure to the EWE by asking how many times each weather event (asked about in the prior question) was experienced	Categories: 1 EWE type, once; 1 EWE type, >once; >1 EWE type, each once; and >1 EWE type of EWE, at least 1 > once
Traumatic exposure to EWE	We assessed intensity of EWE-related stressors using 7 items from the TESS; we used the full 6-item ‘resource loss/being in need’ subscale and 1 item from the ‘damage to home and goods’ subscale	We summed total scores; used dichotomous scores for single items

### Analysis

The aim of our longitudinal analyses was to examine changes in resource insecurities and sexual and mental health outcomes across two time points: at baseline, during a period of heavy rains/flooding; and at the 6-month follow-up, under dry conditions. We first conducted descriptive statistics including calculating means/standard deviations for continuous outcome variables and proportions for categorical outcome variables. We then built generalized estimating equation (GEE) models with an unstructured correlation matrix to estimate differences over time in resource insecurity outcomes (n=4), sexual health outcomes (n=3), and mental health outcomes (n=2), accounting for repeated measures within participants.^37^ Linear GEE models (identity link) were applied for continuous outcomes (e.g. depression scores), while logistic GEE models (logit link) were used for binary outcomes (e.g. transactional sex). All longitudinal models were adjusted for key sociodemographic covariates measured at baseline, including age, education level, employment status, relationship status, and motherhood. Baseline values of outcome variables were also included to account for initial differences and to better isolate the association with time point. We also conducted sensitivity analyses to adjust for EWE exposure.

We examined three variables (motherhood and two TESS indicators) as moderators of the relationships between time point and each of the nine outcomes (27 interactions). For this interaction analysis, the full dataset was used, as motherhood was imbalanced across locations and, when stratified by location, collinearity was observed between motherhood and TESS that could bias the estimates. Using the full dataset allowed stable estimation of interaction effects between time point and potential moderators. Due to high collinearity between the TESS score and time point in longitudinal analyses, we could not include the overall TESS score as an interaction term. To avoid model instability, two TESS items from the ‘resource loss/being in need’ subscale [‘Because of the EWE did you have to spend the night somewhere other than in your home?’ (displacement); ‘Did you need help getting water and food during the weather event?’ (need resource assistance)]^36^ were assessed as moderators; items were selected based on evidence documenting the harms of flood-related displacement^14,38^ and psychosocial stressors of requiring assistance to meet survival needs.^39,40^

Adjusted odds ratios (aOR) were reported for binary outcomes and adjusted beta coefficients (aβ) were reported for continuous outcomes, along with 95% confidence intervals and corresponding *P* values. Separate GEE models were fit for participants from Nairobi and Kisumu to explore potential site-specific differences in associations. Baseline (heavy rains/flooding) measures served as the reference time point for all comparisons. Given the number of outcomes assessed and absence of formal multiplicity adjustment, these analyses should be considered exploratory and hypothesis generating; accordingly, all findings should be interpreted cautiously due to increased potential for Type I error.^41,42^ Effect sizes (ORs for dichotomous outcomes, Cohen’s d for continuous outcomes^43^) and confidence intervals are reported alongside *P* values to support interpretation of the magnitude and precision of observed associations, and borderline significant results are interpreted cautiously. Analyses were conducted using Stata SE version 18.0 (STATA Corp., TX, USA) and statistical significance was determined using a two-sided alpha of 0.05.

### Study ethics

Ethical approval was obtained from the University of Toronto Research Ethics Board (43514), Amref Ethics and Scientific Review Committee (ESRC P1408/2023), and Kenya’s National Commission for Science, Technology, and Innovation (NACOSTI). Written informed consent was obtained from all participants aged ≥16 years. Participants were informed that no identifying information would be linked to their responses and that data access was limited to authorized study staff. Each participant received an honorarium of 900 Kenyan Shillings (∼$7 USD), determined collaboratively with Kenyan community partners to reflect participation costs (e.g. travel, childcare) considering local socioeconomic factors. Consistent with evidence recognizing adolescents aged 16–17 years as capable of providing informed consent for health research,^44,45^ and Kenya’s national HIV testing guidelines (youth aged ≥15 years can test independently^46^), parental consent was not required. Trained social workers from community collaborators (Elim Trust, CSA) provided on-site support and referrals as needed. Adolescents were meaningfully involved in study design, participant recruitment, and data collection as peer researchers throughout implementation.^45^

## Results

### Participant characteristics

The study sample included 597 participants at baseline: 249 (41.7%) from Nairobi and 348 (58.3%) from Kisumu; baseline differences by site are presented in Table 2. The mean age of participants was 20.13 (SD 2.5) years, with Nairobi participants significantly older than Kisumu participants (21.15 vs 19.39 years, *P* < .001). Parenthood status also differed between sites (*P* < .001); nearly all participants in Nairobi (n=244, 98.0%) reported having children compared with 37.4% (n=130) in Kisumu; this was a feature of the study design.

**Table 2:** Baseline characteristics of AGYW participants in the ‘Rada Ya Weather’ cohort study, Nairobi and Kisumu, Kenya (N=597).

Participant characteristics	Total sample N (%)	Nairobi (n=249, 41.7%)	Kisumu (n=348,58.3%)	*P*-value
Age (mean, SD)	20.13 (2.5)	21.15 (2.15)	19.39 (2.49)	<.001
Education				.215
Less than secondary	384 (64.3)	153 (61.4)	231 (66.4)	
Completed secondary school or higher	213 (35.7)	96 (38.6)	117 (33.6)	
Employment status				<.001
Employed (full/part-time)	79 (13.2)	63 (25.3)	16 (4.6)	
Unemployed/student	518 (86.8)	186 (74.7)	332 (95.4)	
Relationship status				<.001
No current partner	172 (29.1)	92 (37.0)	80 (23.3)	
Married	79 (13.3)	31 (12.4)	48 (13.9)	
Dating	327 (55.1)	114 (45.8)	213 (61.9)	
Casual dating/multiple partners	15 (2.5)	12 (4.8)	3 (0.9)	
Motherhood (have children)				<.001
No	223 (37.4)	5 (2.0)	218 (62.6)	
Yes	374 (62.6)	244 (98.0)	130 (37.4)	
Number of extreme weather types experienced in last year				<.001
1	97 (16.2)	4 (1.6)	93 (26.7)	
2–4	310 (51.9)	90 (36.1)	220 (63.2)	
5+	190 (31.8)	155 (62.3)	35 (10.1)	
Extreme weather occurrence by type				
Extreme rain/flooding	511(85.6)	230 (92.4)	281 (80.8)	<.001
Changes in expected rain patterns in a season	371 (62.1)	215 (86.4)	156 (44.8)	<.001
Changes in expected temperature (heat, cold)	359 (60.1)	182 (73.1)	177 (50.9)	<.001
Drought	192 (32.2)	92 (36.9)	100 (28.7)	.034
Extreme heat	257 (43.0)	214 (85.9)	43 (12.4)	<.001
Extreme wind	113 (18.9)	70 (28.1)	43 (12.4)	<.001
Extreme cold	212 (35.5)	153 (61.4)	59 (16.9)	<.001
Fire (home fire, wildfire)	122 (20.4)	114 (45.8)	8 (2.3)	<.001
Storm (e.g. hurricane)	21 (3.5)	7 (2.8)	14 (4.0)	.428
Landslide	15 (2.5)	4 (1.6)	11 (3.2)	.231
Earthquake	5 (0.8)	0	5 (1.4)	.058
Frequency of EWEs in last year				.001
1 type of weather, once in last year	37 (6.2)	1 (0.4)	36 (10.4)	
1 type of weather, more than once in last year	60 (10.0)	3 (1.2)	57 (16.4)	
more than 1 weather type, all only once in the last year	22 (3.7)	17 (6.8)	5 (1.4)	
more than 1 type, at least 1 occurred more than once in last year	478 (80.1)	228 (91.6)	250 (71.8)	
Time 2: TESS indicators (Yes)				
Did you have to spend the night somewhere other than in your home?	391 (65.5%)	171 (68.7%)	220 (63.2%)	.166
Did you need help getting water and food during the weather event? (n=2)	225 (37.8%)	146 (58.6%)	79 (22.8%)	<.001
Did you need help getting clothing during the weather event? (n=1)	321 (53.9%)	146 (58.6%)	175 (50.4%)	.047
Did you need a place to stay (away from your home) after the weather event? (n=2)	262 (44.0%)	154 (61.9%)	108 (31.2%)	<.001
Did you or your caregivers suffer financially because of the weather event? (n=3)	415 (69.9%)	214 (85.9%)	201 (58.3%)	<.001
Did you or your caregivers need financial help from others because of hardships caused by the weather event? (n=2)	392 (65.9%)	197 (79.1%)	195 (56.4%)	<.001
Was your home damaged in the weather event? (n=6)	324 (54.8%)	176 (70.7%)	148 (43.3%)	<.001

n: number of missing cases.

Cumulative exposure to EWEs over the past year differed significantly between sites (*P* < .001). In Nairobi, a higher proportion of participants reported experiencing ≥5 types of EWEs (n=155, 62.3%), while in Kisumu, most reported exposure to 2–4 types (n=220, 63.2%). Regarding EWE frequency, Nairobi participants were more likely to report multiple types of EWEs, with at least one type occurring more than once during the past year (91.6% in Nairobi vs 71.8% in Kisumu, *P* = .001).

### Changes in resource insecurity and mental and sexual health outcomes between time points

At the 6-month follow-up, 586 participants completed the survey, yielding a high retention rate (98.2%). Given the minimal loss to follow-up, longitudinal analyses were conducted using a complete case analysis approach, which is appropriate when the proportion of missing data is low and unlikely to bias results.^47^ Changes in study outcomes from baseline (Time 1 [T1], heavy rains/flooding) to follow-up (Time 2 [T2], dry conditions) revealed both shared and distinct patterns in Nairobi and Kisumu, presented in Table 3 and detailed below. Sensitivity analyses that additionally adjusted for EWE exposure yielded consistent results (Supplementary Table S1).

**Table 3:** Longitudinal analysis of seasonal changes in resource insecurity, sexual and mental health outcomes among AGYW participants in the ‘Rada Ya Weather’ cohort study, Nairobi and Kisumu, Kenya (N=586).

		Nairobi				Kisumu			
Outcome	Time period	aOR/β	95% CI	*P*-value	Cohen’s d	aOR/aβ	95% CI	*P*-value	Cohen’s d
**Resource insecurity (higher scores reflect higher insecurity) (reference = heavy rains and flooding season)**									
** Water insecurity (0=secure, 1=insecure) (adjusted odds ratio)**									
	Follow-up (dry season)	0.25	0.07, 0.86	.028	n/a	1.26	0.64, 2.46	.501	n/a
** Food insecurity (aβ)**									
	Follow-up (dry season)	−2.89	−4.74, −1.04	.002	−0.30	−2.68	−3.84, −1.53	<.001	−0.30
** Sanitation insecurity (aβ)**									
	Follow-up (dry season)	−3.14	−6.18, −0.09	.043	−0.33	−2.18	−4.18, −0.18	.032	−0.20
** Menstruation insecurity (aβ)**									
	Follow-up (dry season)	−1.03	−2.46, 0.40	.158	−0.28	0.69	−0.67, 2.06	.318	0.03
**Sexual health (reference = heavy flooding season)**									
** CUSE (aβ)**									
	Follow-up (dry season)	−1.30	−2.19, −0.41	.004	−0.35	1.05	0.42, 1.67	.001	0.23
** Transactional sex engagement (aOR)**									
	Follow-up (dry season)	0.17	0.03, 0.82	.028	n/a	0.13	0.05, 0.31	<.001	n/a
** SRP (aβ)**									
	Follow-up (dry season)	2.38	0.35, 4.41	.022	0.21	2.13	0.54, 3.72	.009	0.23
** Mental health (reference = heavy flooding season)**									
** Depression (aβ)**									
	Follow-up (dry season)	−2.89	−4.78, −0.99	.003	−0.36	1.30	0.24, 2.36	.016	0.13
** Eco-anxiety (aβ)**									
	Follow-up (dry season)	−1.91	−4.41, 0.59	.135	−0.29	−2.19	−3.39, −0.99	<.001	−0.26

Intervention (season) effect calculated using GEE linear/logistic regression model with an unstructured correlation matrix. Adjusted intervention (season) effect, controlling covariates (age, education, employment change, relationship status, motherhood), and baseline scores.

#### Changes in resource insecurity outcomes

**Figure 1:**
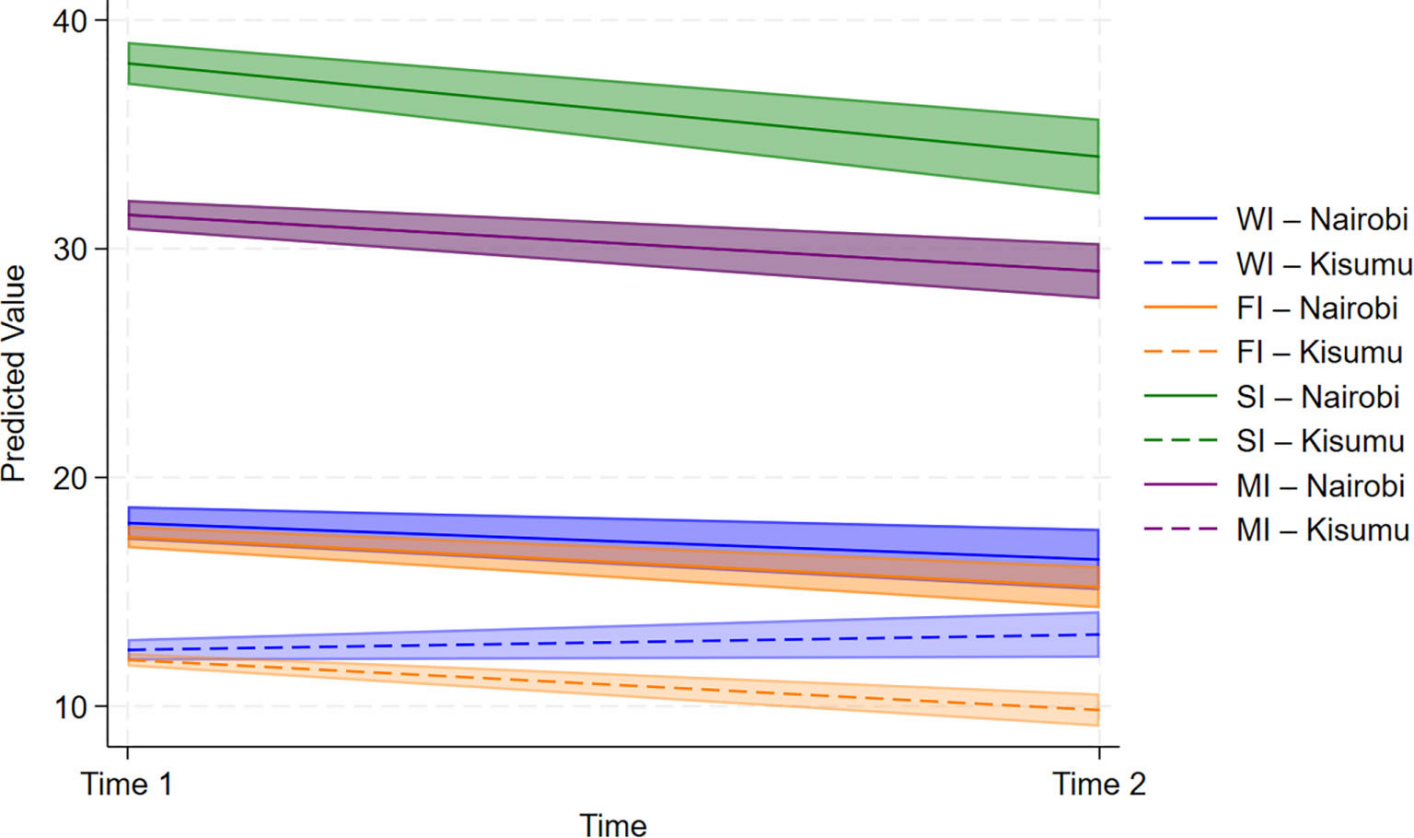
Longitudinal changes in resource insecurity outcomes between timepoints among adolescent girl and young women participants in the ‘Rada Ya Weather’ cohort study, Nairobi and Kisumu, Kenya (N=597) WI-Nairobi: Water insecurity scores in Nairobi. WI-Kisumu: Water insecurity scores in Kisumu. FI-Nairobi: Food insecurity scores in Nairobi. FI-Kisumu: Food insecurity scores in Kisumu. SI-Nairobi: Sanitation insecurity scores in Nairobi. SI-Kisumu: Sanitation insecurity scores in Kisumu. MI-Nairobi: Menstruation insecurity scores in Nairobi. MI-Kisumu: Menstruation insecurity scores in Kisumu.

Participants in Nairobi reported a significant decrease in water insecurity (WI) at T2 compared with T1 (aOR=0.25, 95% CI: 0.07 to 0.86, *P* = .028, large effect). In Kisumu, there were no significant differences in WI reported between T1 and T2 (aOR=1.26, 95% CI: 0.64 to 2.46, *P* = .501, small effect). Food insecurity (FI) was lower at T2 than at T1 in both sites (Nairobi: aβ= −2.89, 95% CI: −4.74 to −1.04, *P* = .002, Cohen’s d= −0.30, small effect; Kisumu: aβ= −2.68, 95% CI: −3.84 to −1.53, *P* < .001, Cohen’s d= −0.30, small effect). Similarly, sanitation insecurity (SI) was significantly lower at T2 compared with T1 in both Nairobi (aβ= −3.14, 95% CI: −6.18 to −0.009, *P* = .043, Cohen’s d= −0.33, small effect) and Kisumu (aβ= −2.18, 95% CI: −4.18 to −0.18, *P* = .032, Cohen’s d= −0.33, small effect). There were no significant differences in menstruation insecurity between time points in either site. These changes are illustrated in Fig. 1.

As detailed in Table 4, the interaction between time point, resource insecurity, and motherhood was significant; compared with non-mothers at T1 (heavy rains/flooding), both mothers and non-mothers at T2 had lower FI and SI. There were also significant interactions between time point, resource insecurity, and TESS indicators: (i) compared with non-displaced T1 participants, participants with T1 displacement reported higher FI and SI; (ii) compared with non-displaced T1 participants, non-displaced participants at T2 had lower FI and SI; (iii) compared with T1 participants needing no resource assistance, those who needed resource assistance at T1 had higher WI, FI, and SI; and (iv) among participants not needing resource assistance, T2 participants had lower FI and SI than T1 participants.

**Table 4:** Longitudinal interaction analysis of seasonal changes in resource insecurity, sexual health and mental health outcomes among AGYW in the ‘Rada Ya Weather’ cohort study, Nairobi and Kisumu, Kenya (N=586).

		Whole data (adjusted)		
Outcome	Time period	aOR/β	95% CI	*P*-value
**Resource insecurity (higher scores reflect higher insecurity) [reference=heavy rains and flooding season]**				
**Water insecurity (0=secure, 1=insecure) (OR)**				
**Time X Motherhood**				
Time 1: Heavy rains and flooding season #No (not a mother)		Ref.		
Time 1: Heavy rains and flooding season #Yes (a mother)		1.28	(0.88, 1.85)	.200
Time 2: Dry season #No (not a mother)		1.07	(0.59, 1.92)	.811
Time 2: Dry season #Yes (a mother)		1.16	(0.69, 1.96)	.574
**Time X Traumatic Exposure Severity Indicator: displacement**				
Time X Did you have to spend the night somewhere other than in your home?				
Time 1: Heavy rains and flooding season #No (not displaced)		Ref.		
Time 1: Heavy rains and flooding season #Yes (displaced)		1.28	0.98, 1.67	.066
Time 2: Dry season #No (not displaced)		1.31	0.84, 2.05	.228
Time 2: Dry season #Yes (displaced)		0.63	0.37, 1.10	.105
**Time X Traumatic Exposure Severity Indicator: needed resource assistance**				
Time X Did you need help getting water and food during the weather event?				
Time 1: Heavy rains and flooding season #No (no assistance needed)		Ref.		
Time 1: Heavy rains and flooding season #Yes (assistance needed)		1.43	1.14, 1.79	.002
Time 2: Dry season #No (no assistance needed)		0.83	0.50, 1.37	.471
Time 2: Dry season #Yes (assistance needed)		1.60	1.00, 2.54	.050
**Food insecurity (aβ)**				
**Time X Motherhood**				
Time 1: Heavy rains and flooding season #No (not a mother)		Ref.		
Time 1: Heavy rains and flooding season #Yes (a mother)		−0.05	−0.71, 0.61	.887
Time 2: Dry season #No (not a mother)		−2.10	−3.16, −1.05	<.001
Time 2: Dry season #Yes (a mother)		−1.76	−2.78, −0.73	.001
**Time X Traumatic Exposure Severity Indicator: displacement**				
Time X Did you have to spend the night somewhere other than in your home?				
Time 1: Heavy rains and flooding season #No (not displaced)		Ref.		
Time 1: Heavy rains and flooding season #Yes (displaced)		0.53	0.06, 0.99	.028
Time 2: Dry season #No (not displaced)		−2.46	−3.27, −1.65	<.001
Time 2: Dry season #Yes (displaced)		0.16	−0.93, 1.25	.774
**Time X Traumatic Exposure Severity Indicator: needed resource assistance**				
Time X Did you need help getting water and food during the weather event?				
Time 1: Heavy rains and flooding season #No (no assistance needed)		Ref.		
Time 1: Heavy rains and flooding season #Yes (assistance needed)		0.53	0.14, 0.93	.008
Time 2: Dry season #No (no assistance needed)		−3.56	−4.46, −2.66	<.001
Time 2: Dry season #Yes (assistance needed)		−0.31	−1.18, 0.57	.492
**Sanitation insecurity (aβ)**				
**Time X Motherhood**				
Time 1: Heavy rains and flooding season #No (not a mother)			Ref	
Time 1: Heavy rains and flooding season #Yes (a mother)		−0.05	−1.36, 1.25	.935
Time 2: Dry season #No (not a mother)		−4.03	−5.97, −2.10	<.001
Time 2: Dry season #Yes (a mother)		−1.73	−3.61, 0.15	.071
**Time X Traumatic Exposure Severity Indicator: displacement**				
Time X Did you have to spend the night somewhere other than in your home?				
Time 1: Heavy rains and flooding season #No (not displaced)			Ref.	
Time 1: Heavy rains and flooding season #Yes (displaced)		1.09	0.16, 2.01	.021
Time 2: Dry season #No (not displaced)		−2.73	−4.21, −1.24	<.001
Time 2: Dry season #Yes (displaced)		−1.34	−3.34, 0.67	.191
**Time X Traumatic Exposure Severity Indicator: needed resource assistance**				
Time X Did you need help getting water and food during the weather event?				
Time 1: Heavy rains and flooding season #No (no assistance needed)			Ref.	
Time 1: Heavy rains and flooding season #Yes (assistance needed)		1.34	0.56, 2.12	.001
Time 2: Dry season #No (no assistance needed)		−4.54	−6.38, −2.70	<.001
Time 2: Dry season #Yes (assistance needed)		−0.20	−1.68, 1.28	.791
**Menstruation insecurity (aβ)**				
Time X Motherhood		Omitted due to collinearity		
**Sexual health [reference=heavy rains/flooding season]**				
**CUSE (aβ)**				
**Time X Motherhood**				
Time 1: Heavy rains and flooding season #No (not a mother)		Ref.		
Time 1: Heavy rains and flooding season #Yes (a mother)		0.74	0.31, 1.16	.001
Time 2: Dry season #No (not a mother)		0.61	−0.10, 1.32	.091
Time 2: Dry season #Yes (a mother)		0.39	−0.15, 0.93	.155
**Time X Traumatic Exposure Severity Indicator: displacement**				
Time X Did you have to spend the night somewhere other than in your home?				
Time 1: Heavy rains and flooding season #No (not displaced)		Ref.		
Time 1: Heavy rains and flooding season #Yes (displaced)		0.07	−0.27,0.41	.690
Time 2: Dry season #No (not displaced)		0.21	−0.29, 0.72	.410
Time 2: Dry season #Yes (displaced)		−0.22	−0.89, 0.46	.532
**Time X Traumatic Exposure Severity Indicator: needed resource assistance**				
Time X Did you need help getting water and food during the weather event?				
Time 1: Heavy rains and flooding season #No (no assistance needed)		Ref.		
Time 1: Heavy rains and flooding season #Yes (assistance needed)		0.09	−0.20, 0.40	.513
Time 2: Dry season #No (no assistance needed)		0.32	−0.29, 0.94	.304
Time 2: Dry season #Yes (assistance needed)		−0.01	−0.53, 0.52	.982
**Transactional sex engagement (aOR)**				
**Time X Motherhood**				
Time 1: Heavy rains and flooding season #No (not a mother)		Ref.		
Time 1: Heavy rains and flooding season #Yes (a mother)		0.85	0.57, 1.26	.414
Time 2: Dry season #No (not a mother)		0.16	0.09, 0.30	<.001
Time 2: Dry season #Yes (a mother)		0.21	0.12, 0.38	<.001
**Time X Traumatic Exposure Severity Indicator: displacement**				
Time X Did you have to spend the night somewhere other than in your home?				
Time 1: Heavy rains and flooding season #No (not displaced)		Ref.		
Time 1: Heavy rains and flooding season #Yes (displaced)		0.96	0.73, 1.26	.773
Time 2: Dry season #No (not displaced)		0.16	0.09, 0.26	<.001
Time 2: Dry season #Yes (displaced)		0.36	0.18, 0.68	.002
**Time X Traumatic Exposure Severity Indicator: needed resource assistance**				
Time X Did you need help getting water and food during the weather event?				
Time 1: Heavy rains and flooding season #No (no assistance needed)		Ref.		
Time 1: Heavy rains and flooding season #Yes (assistance needed)		1.33	1.04, 1.70	.020
Time 2: Dry season #No (no assistance needed)		0.12	0.06, 0.23	<.001
Time 2: Dry season #Yes (assistance needed)		0.35	0.21, 0.59	<.001
**SRP (aβ)**				
**Time X Motherhood**				
Time 1: Heavy rains and flooding season #No (not a mother)		Ref.		
Time 1: Heavy rains and flooding season #Yes (a mother)		−0.42	−1.37, 0.53	.386
Time 2: Dry season #No (not a mother)		0.96	−0.62, 2.54	.233
Time 2: Dry season #Yes (a mother)		2.83	1.56, 4.10	<.001
**Time X Traumatic Exposure Severity Indicator: displacement**				
Time X Did you have to spend the night somewhere other than in your home?				
Time 1: Heavy rains and flooding season #No (not displaced)		Ref.		
Time 1: Heavy rains and flooding season #Yes (displaced)		−0.61	−1.24, 0.03	.060
Time 2: Dry season #No (not displaced)		2.37	1.25, 3.49	<.001
Time 2: Dry season #Yes (displaced)		1.27	−0.21, 2.75	.092
**Time X Traumatic Exposure Severity Indicator: needed resource assistance**				
Time X Did you need help getting water and food during the weather event?				
Time 1: Heavy rains and flooding season #No (no assistance needed)		Ref.		
Time 1: Heavy rains and flooding season #Yes (assistance needed)		−0.39	−0.98, 0.20	.195
Time 2: Dry season #No (no assistance needed)		2.99	1.66, 4.32	<.001
Time 2: Dry season #Yes (assistance needed)		1.40	0.24, 2.57	.019
**Mental health [reference=heavy rains/flooding season]**				
**Depression (aβ)**				
**Time X Motherhood**				
Time 1: Heavy rains and flooding season #No (not a mother)		Ref.		
Time 1: Heavy rains and flooding season #Yes (a mother)		0.73	0.04, 1.42	.039
Time 2: Dry season #No (not a mother)		0.51	−0.57, 1.60	.353
Time 2: Dry season #Yes (a mother)		0.32	−0.69, 1.32	.536
**Time X Traumatic Exposure Severity Indicator: displacement**				
Time X Did you have to spend the night somewhere other than in your home?				
Time 1: Heavy rains and flooding season #No (not displaced)		Ref.		
Time 1: Heavy rains and flooding season #Yes (displaced)		0.55	0.05, 1.05	.031
Time 2: Dry season #No (not displaced)		0.32	−0.49, 1.13	.439
Time 2: Dry season #Yes (displaced)		−0.13	−1.16, 0.91	.809
**Time X Traumatic Exposure Severity Indicator: needed resource assistance**				
Time X Did you need help getting water and food during the weather event?				
Time 1: Heavy rains and flooding season #No (no assistance needed)		Ref.		
Time 1: Heavy rains and flooding season #Yes (assistance needed)		0.59	0.18, 1.00	.005
Time 2: Dry season #No (no assistance needed)		−0.70	−1.68, 0.27	.158
Time 2: Dry season #Yes (assistance needed)		1.03	0.17, 1.88	.019
**Eco-anxiety (aβ)**				
**Time X Motherhood**				
Time 1: Heavy rains and flooding season #No (not a mother)		Ref.		
Time 1: Heavy rains and flooding season #Yes (a mother)		−0.13	−0.98, 0.72	.765
Time 2: Dry season #No (not a mother)		−1.96	−3.21,-0.70	.002
Time 2: Dry season #Yes (a mother)		−2.19	−3.37, −1.00	.000
**Time X Traumatic Exposure Severity Indicator: displacement**				
Time X Did you have to spend the night somewhere other than in your home?				
Time 1: Heavy rains and flooding season #No (not displaced)		Ref .		
Time 1: Heavy rains and flooding season #Yes (displaced)		0.58	−0.04, 1.21	.069
Time 2: Dry season #No (not displaced)		−2.41	−3.36, −1.45	<.001
Time 2: Dry season #Yes (displaced)		−0.24	−1.71, 1.23	.746
**Time X Traumatic Exposure Severity Indicator: needed resource assistance**				
Time X Did you need help getting water and food during the weather event?				
Time 1: Heavy rains and flooding season #No (no assistance needed)		Ref.		
Time 1: Heavy rains and flooding season #Yes (assistance needed)		1.22	0.67, 1.76	<.001
Time 2: Dry season #No (no assistance needed)		−3.18	−4.36, −1.99	<.001
Time 2: Dry season #Yes (assistance needed)		0.04	−1.01, 1.08	.947

Intervention (season) effect calculated using GEE linear/logistic regression model with an unstructured correlation matrix. Adjusted intervention (season) effect, controlling covariates (location, age, education, employment change, relationship status, motherhood), and baseline scores.

#### Changes in sexual health outcomes

Engagement in transactional sex (TS) was lower at T2 compared with T1 in both Nairobi (aOR=0.17, 95% CI: 0.03 to 0.82, *P* = .028, large effect) and Kisumu (aOR=0.13, 95% CI: 0.05 to 0.31, *P* < .001, large effect) (Table 3). As reported in Table , there was a significant interaction between time point, TS, and motherhood; compared with non-mothers at T1, the odds of TS were significantly lower at T2 for both non-mothers and mothers. There were also significant interactions with TESS indicators: (i) compared with non-displaced participants at T1, at T2 both non-displaced and displaced participants had lower odds of TS; (ii) among T1 participants, those needing resource assistance had higher odds of TS vs those who did not; and (iii) compared with participants needing no resource assistance at T1, at T2 TS was lower among both participants who needed and did not need resource assistance.

SRP scores were higher at T2 compared with T1 in both Nairobi (aβ=2.38, 95% CI: 0.35 to 4.41, *P* = .022, Cohen’s d= 0.21, small effect) and Kisumu (aβ=2.13, 95% CI: 0.54 to 3.72, *P* = .009, Cohen’s d=0.23, small effect). There was a significant interaction with motherhood; compared with non-mothers at T1, SRP was significantly higher at T2 among mothers. There were also significant interactions with TESS indicators: (i) among non-displaced participants, participants at T2 had higher SRP than at T1; and (ii) compared with T1 participants not needing resource assistance, T2 participants had higher SRP, regardless of whether needing resource assistance or not.

**Figure 2:**
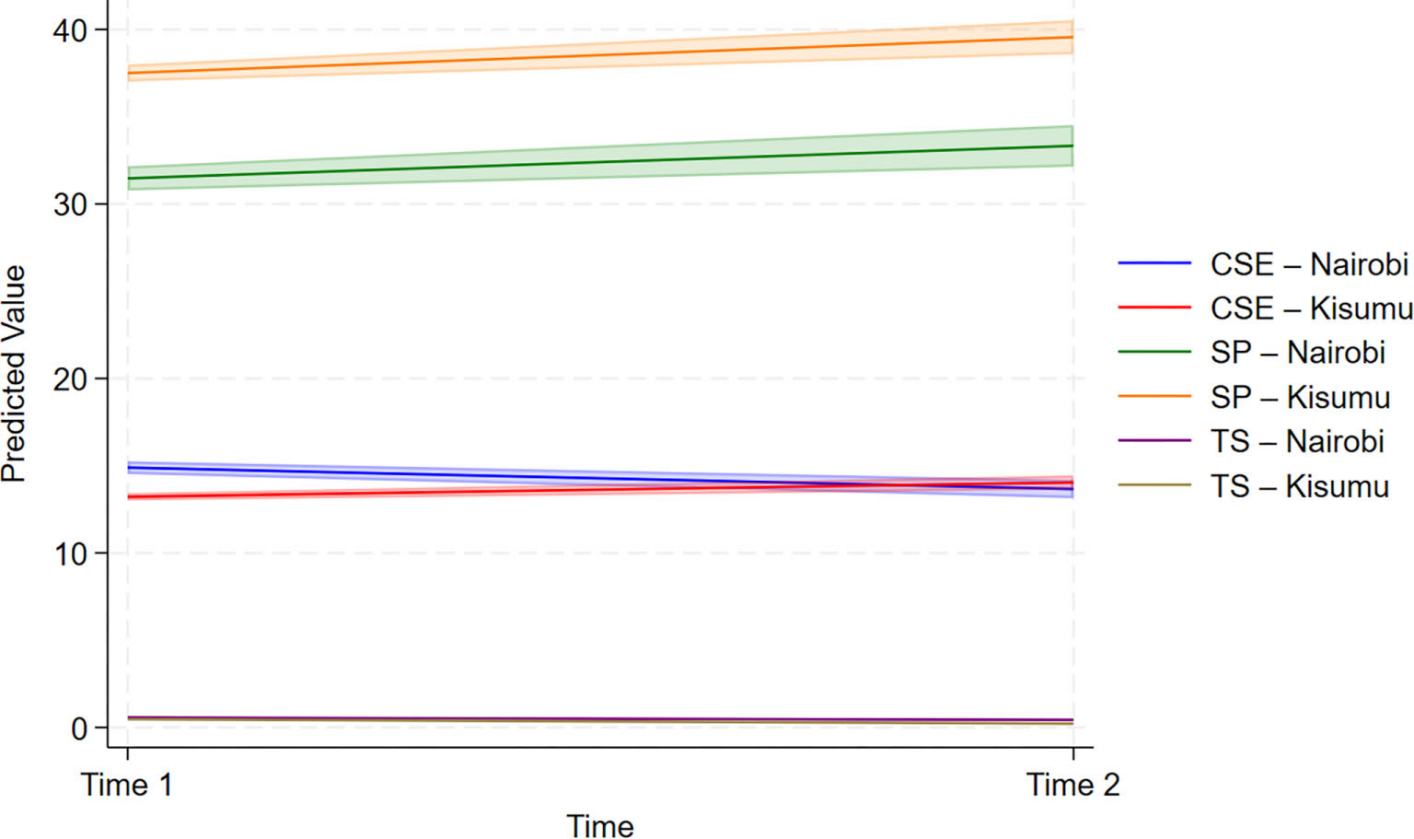
Longitudinal changes in sexual health outcomes between timepoints among adolescent girl and young women participants in the ‘Rada Ya Weather’ cohort study, Nairobi and Kisumu, Kenya (N=597) CSE-Nairobi: Condom use self-efficacy scores in Nairobi. CSE-Kisumu: Condom use self-efficacy scores in Kisumu. SP-Nairobi: Sexual relationship power scores in Nairobi. SP-Kisumu: Sexual relationship power scores in Kisumu. TS-Nairobi: Transactional sex in Nairobi. TS-Kisumu: Transactional sex in Kisumu.

Compared with T1, CUSE was significantly lower in Nairobi at T2 (aβ= −1.30, 95% CI: −2.19 to −0.41, *P* = .004, Cohen’s d= −0.35, small effect), while it was significantly higher in Kisumu (aβ=1.05, 95% CI: 0.42 to 1.67, *P* = .001, Cohen’s d= 0.23, small effect). There was a significant interaction with motherhood, whereby at T1, mothers had significantly higher CUSE compared with non-mothers. These changes over time are depicted in Fig. 2.

#### Changes in mental health outcomes

Eco-anxiety scores were lower at T2 (dry conditions) compared with T1 (heavy rains/flooding) at both sites, with a statistically significant reduction observed in Kisumu (aβ= −2.19, 95% CI: −3.39 to −0.99, Cohen’s d= −0.26, small effect) and a non-significant downward trend in Nairobi (aβ= −1.91, 95% CI: −4.41 to 0.59, Cohen’s d= −0.29, small effect). Compared with non-mothers at T1, at T2 eco-anxiety was significantly lower among both mothers and non-mothers. There were also significant interactions with TESS indicators: (i) among non-displaced participants, T2 participants had lower eco-anxiety scores compared with T1 participants; (ii) among T1 participants, those who needed resource assistance had higher eco-anxiety scores compared with those not needing resource assistance; and (iii) among participants not needing resource assistance, eco-anxiety was lower at T2 compared with T1.

**Figure 3:**
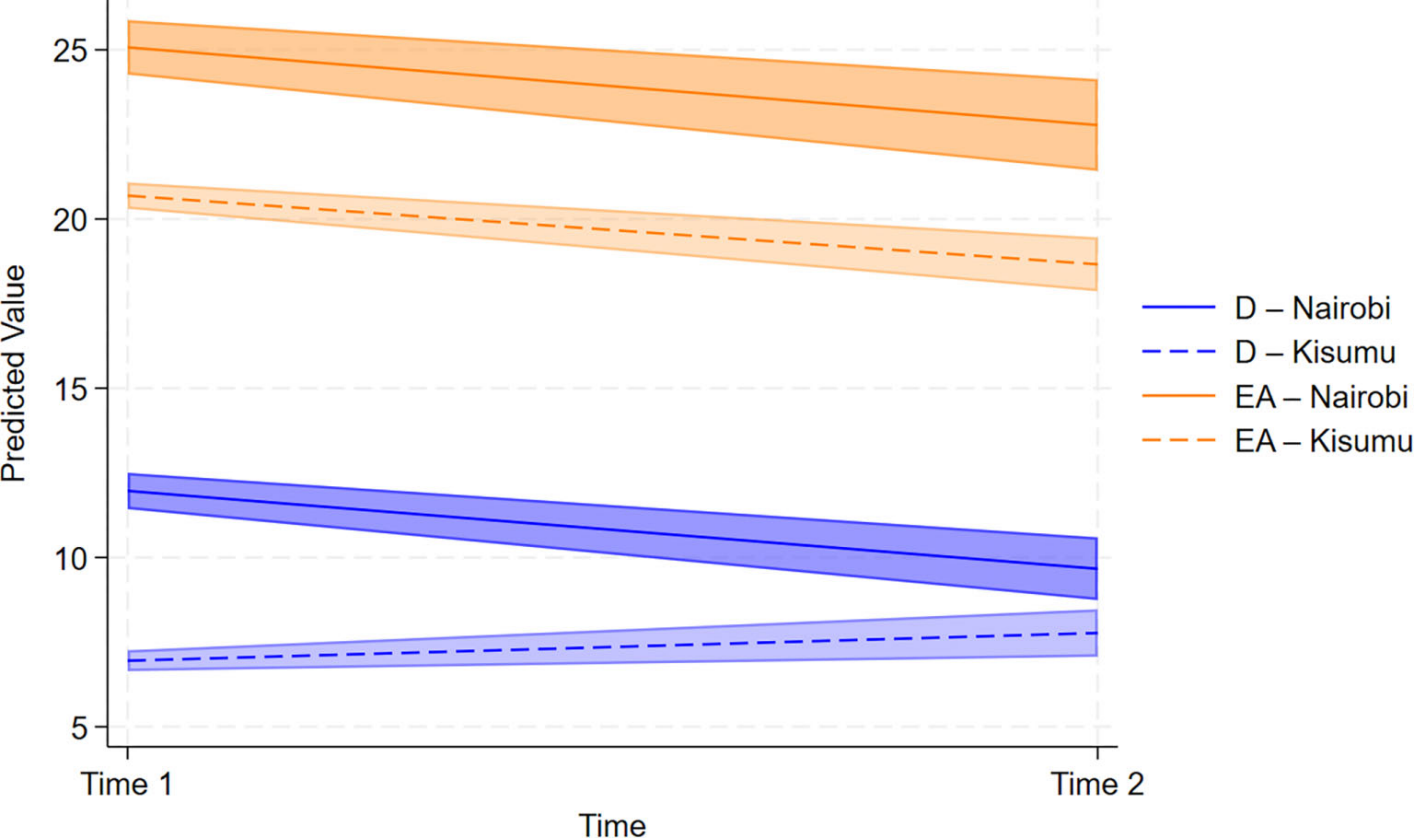
Longitudinal changes in mental health outcomes between timepoints among adolescent girl and young women participants in the ‘Rada Ya Weather’ cohort study, Nairobi and Kisumu, Kenya (N=597) D-Nairobi: Depression scores in Nairobi. D-Kisumu: Depression scores in Kisumu. EA-Nairobi: Ecoanxiety scores in Nairobi. EA-Kisumu: Ecoanxiety scores in Kisumu.

Depression symptoms were significantly lower in Nairobi at T2 compared with T1 (aβ= −2.89, 95% CI: −4.78 to −0.99, Cohen’s d= −0.36, small effect). By contrast, depression symptoms were higher in Kisumu at T2 compared with T1 (aβ=1.30, 95% CI: 0.24 to 2.36, Cohen’s d= 0.13, small effect). These changes are illustrated in Fig. 3.

There was an interaction between time point, motherhood, and depression, whereby at T1, mothers had higher depression scores compared with non-mothers. There were also interactions with TESS indicators: (i) at T1, participants who were displaced had higher depression scores compared with those who were not displaced; (ii) at T1, participants who needed assistance had higher depression scores compared with those not needing assistance; and (iii) participants at T2 who needed resource assistance had higher depression scores compared with those not needing resource assistance at T1.

## Discussion

Our longitudinal study assessed differences in resource security, sexual health, and mental health across two time points among a sample of AGYW in Nairobi’s informal settlements and Kisumu’s fishing communities. Compared with baseline, we observed lower levels of food, water, and sanitation insecurity, transactional sex, and eco-anxiety, alongside higher SRP at follow-up. Regional differences may reflect the unequal flood impact, with Nairobi county experiencing greater damage.^2,48^ These findings contribute novel evidence on AGYW’s EWE- and resource insecurity-related vulnerabilities in Kenya with implications for gender, age, and contextually tailored climate adaptation and health interventions.

We found lower reported resource insecurity at follow-up compared with baseline, particularly in Nairobi. This pattern is consistent with research documenting the destructive effects of Kenya’s flooding and heavy rains on agricultural production that increases food insecurity,^2,3,5^ and on water^3^ and sanitation^14^ hygiene (WASH) infrastructure that elevates WASH insecurities. Higher resource insecurities among participants who experience displacement and need resource assistance suggest that climate shocks may aggravate pre-existing vulnerabilities. While Kisumu participants reported greater food and sanitation security, water insecurity persisted. This may reflect vulnerabilities in fishing communities to climate-related EWEs that can reduce access to and availability of natural and non-contaminated water.^13,14^ Other Kenyan studies have noted increased water insecurity in the dry season due to water shortages that reduce availability and increase the price of water.^12^ Menstruation insecurity did not vary between time points, potentially reflecting persistent structural and sociocultural barriers to realizing optimal menstrual health, including limited product affordability, inadequate WASH infrastructure, and enduring stigma that are less sensitive to short-term environmental change.^49–52^ This persistence highlights the importance of addressing menstrual health as an ongoing, rather than crisis-driven, concern. Future time-series research could investigate if these differences we noted over time are caused by seasonal changes in Kisumu’s water insecurity and could focus on specific water security dimensions (e.g. reliability, safety). Together, the findings underscore the potential need to strengthen food, WASH, and menstrual health programmes^3,14^ as part of climate adaptation strategies among AGYW in EWE-affected settings.^13^

We found lower reported transactional sex and higher SRP at follow-up in Kisumu and Nairobi compared with baseline. These findings align with prior research describing linkages between resource insecurity during flooding and survival sex among young women in Kenya and other SSA contexts.^5,8^ The higher odds of transactional sex reported among those needing resource assistance during the heavy rains/flooding further indicates that material insecurities may be associated with sexual risk. Regional variation in CUSE, such as reduced CUSE in Nairobi at follow-up compared with baseline, warrants further exploration. It could, for instance, reflect shifting relationship dynamics, changes in sexual health access, or psychological fatigue following a crisis.^6,9^ Alternatively, increased CUSE in Kisumu may reflect the effectiveness of community-based HIV outreach in this high HIV prevalence setting, post-disaster relief resources, or differentiated social support networks.^17^ Higher CUSE among young mothers may reflect prior engagement with sexual health services. Future qualitative research could explore EWE-related sexual risk and protective factors in each setting.

Our findings highlight that place, timing, and intensity of EWE-related stressors may shape mental health and psychosocial stressors over time in complex ways. Global and regional studies show that repeated or prolonged EWE exposure can have cumulative psychological effects, contributing to elevated anxiety, depression, and suicidality among youth, particularly girls, who often report greater climate-related concern than boys.^53–56^ In our study, eco-anxiety was lower at follow-up compared with baseline in both Kisumu and Nairobi, which may reflect how current exposure to an EWE is linked with higher climate-related distress.^28^ For instance, prior research has described processes of climate numbness or stress habituation.^57^ Eco-anxiety may be more closely linked with periods of heightened environmental stress, and was higher in our study among those needing resource assistance, suggesting that material need insecurities may increase emotional distress linked with the environment. This aligns with systematic review findings noting mental health stressors caused by EWEs in SSA.^11^ Compared with baseline, depression was lower at follow-up in Nairobi while it increased in Kisumu, which may point to differing temporal or contextual dynamics, including but not limited to post-flooding recovery trajectories and psychosocial stressors. However, as these mechanisms of climate numbness, stress habituation, and recovery trajectories were not directly measured in our study, they should be interpreted as hypotheses requiring further investigation. Depression was higher among mothers compared with non-mothers at baseline: this could potentially be due to caregiving-related stressors during EWEs.^58,59^ During the heavy rains/flooding time point, depression was also higher among those needing resource assistance and who had been displaced, again suggesting that traumatic exposure severity indicators of resource loss and being in need may elevate mental health stressors. Our results also signal the need for further longitudinal and mixed-methods mental health EWE-related research with this population of AGYW.^11^

Our study has several limitations. First, its findings are not representative of all AGYW in Kenya; the non-random sample and inclusion criteria requiring participants to have access to a mobile phone and communicate in English, Swahili, or Luo, may have excluded more vulnerable AGYW. Recruitment through peer navigators and community outreach may have introduced selection bias, as participants who were more socially connected were more likely to participate than more vulnerable or hidden AGYW populations. Our population was climate-affected with HIV vulnerabilities, which could limit variability in findings and reduce statistical power. Second, participants may have underreported sexual health practices and mental health symptoms due to social desirability bias, despite confidentiality being maintained throughout data collection. Third, differences in recall periods between baseline and follow-up data collection may have influenced responses and contributed to apparent changes over time. The difference in recall periods for transactional sex (12 months at baseline vs 6 months at follow-up) allowed for interpretation of changes since the previous survey yet may partially account for the observed decline. We used the recall periods recommended in validated scales for each construct (i.e. food, water, sanitation, menstruation insecurities); however, variability in these recall periods may affect temporal alignment across constructs. Dichotomization of water insecurity may have resulted in some loss of information and reduced statistical power; however, this approach was used to align with established thresholds and to enhance interpretability for policy and programming. Fourth, as our study focus is on EWEs at large, we did not assess household and individual-level flooding coping mechanisms or multiple dimensions of flood exposure, sensitivity, and adaptive capacity, which could inform flood preventive actions and mitigation.^3^ Fifth, the findings reflect differences across two time points and should not be interpreted as evidence of causal or seasonal effects. Observed patterns may reflect a combination of seasonal variation, post-flood recovery processes, and other concurrent social or structural changes that cannot be disentangled in this study design. We did not assess potential confounders such as national economic shifts and political influences between the time points, and this is an area for future research. Sixth, while effect sizes were large for changes in transactional sex in both sites and for water insecurity in Nairobi, indicating potentially meaningful shifts, most other observed changes were small, suggesting more modest practical significance despite statistical significance. Additionally, stratified analyses by place accounted for contextual differences yet reduce cross-site comparability. Despite these limitations, the study also has important strengths, including a high retention rate, longitudinal design, inclusion of two climate-affected settings, and the measurement of mental health outcomes, which are understudied in youth EWE-related research in SSA.^11^ We also highlight the potential moderating role of motherhood and TESS in contextualizing experiences of resource insecurity, sexual and mental health.

Our study provides novel longitudinal evidence of differences between two time points in health and well-being among AGYW in two Kenyan regions. Strategies to address intersecting challenges facing AGYW can include youth co-created, EWE-tailored, sexual and mental health programmes that also address underlying resource insecurities. Cross-sectoral frameworks that link climate adaptation, gender equity, and mental and sexual health systems strengthening offer a way forward. Kenya’s national strategies, such as the Climate Change and Health Strategy (2024–2029)^60^ and National Gender and Climate Change Action Plan (2025–2027),^61^ outline coordination mechanisms, and local initiatives like Kisumu’s People’s Plans into Practice,^62^ provide examples of gender-sensitive climate adaptation. Integrating climate, gender, and health programmes can advance equity and resilience among AGYW.^63–65^

## Declaration of Generative AI and AI-assisted technologies in manuscript preparation

None declared.

## Supplementary Material

ihag064_Supplemental_File

## Data Availability

Data will be shared upon reasonable request and obtaining required ethics approvals to the corresponding author.
