# Supplementary material for: Changes in resource insecurity, sexual and mental health among young women after Kenya’s 2024 heavy rains and floods
Source: Int Health. 2026 Jun 24;18(4):522–37. doi: 10.1093/inthealth/ihag064 (PMC13329966; doi:10.1093/inthealth/ihag064)
Supplement: ihag064_Supplemental_File [file ihag064_supplemental_file.docx]

**Supplementary Table 1.** **Longitudinal analysis of seasonal changes in resource insecurity, sexual, and mental health outcomes among adolescent girl and young women participants in the ‘Rada Ya Weather’ cohort study, Nairobi and Kisumu, Kenya adjusted by extreme weather event exposure (N=586)**

| **Outcome** | **Time Period** | **Nairobi** | | | **Kisumu** | | |
| --- | --- | --- | --- | --- | --- | --- | --- |
|  |  | **aOR/β** | **95% CI** | **P-value** | **aOR/aβ** | **95% CI** | **P-value** |
| **Resource insecurity (the higher, the more insecurity)[Reference=** Heavy rains and flooding season] | | | | | | | |
| **Water insecurity (0=Secure, 1= insecure) (adjusted odds ratio)** | | | | |  |  |  |
|  | Follow-up (dry season) | 0.23 | 0.09, 0.58 | 0.002 | 1.46 | 0.94, 2.28 | 0.095 |
| **Food insecurity (aβ) (adjusted beta coefficient)** | | | | |  |  |  |
|  | Follow-up (dry season) | -1.85 | -3.15, -0.55 | 0.005 | -2.17 | -3.06, -1.27 | < 0.001 |
| **Sanitation insecurity (aβ) (adjusted beta coefficient)** | | | |  |  |  |  |
|  | Follow-up (dry season) | -3.32 | -5.63, -1.01 | 0.005 | -2.53 | -4.20, -0.85 | 0.003 |
| **Menstruation insecurity (aβ) (adjusted beta coefficient)** | | | | |  |  |  |
|  | Follow-up (dry season) | -1.32 | -2.72, 0.74 | 0.064 | 0.16 | -0.88, 1.21 | 0.761 |
| **Sexual health [Reference=** Heavy flooding season] | | | | | | | |
| **Condom use self-efficacy (aβ) (adjusted beta coefficient)** | | | | |  |  |  |
|  | Follow-up (dry season) | -1.17 | -2.00, -0.34 | 0.006 | 0.82 | 0.25, 1.38 | 0.005 |
| **Transactional sex engagement (0=no, 1=yes) (adjusted odds ratio)** | | | | |  |  |  |
|  | Follow-up (dry season) | 0.40 | 0.22, 0.74 | 0.003 | 0.14 | 0.08, 0.26 | < 0.001 |
| **Sexual relationship power (aβ) (adjusted beta coefficient)** | | | |  |  |  |  |
|  | Follow-up (dry season) | 1.76 | 0.04, 3.48 | 0.045 | 2.02 | 0.73, 3.30 | 0.002 |
| **Mental health [Reference=** Heavy flooding season] | | | |  |  |  |  |
| **Depression (aβ) (adjusted beta coefficient)** | | | | | | | |
|  | Follow-up (dry season) | -1.97 | -3.34, -0.60 | 0.005 | 0.69 | -0.18, 1.56 | 0.123 |
| **Eco-anxiety (aβ) (adjusted beta coefficient)** | | | | | | | |
|  | Follow-up (dry season) | -1.74 | -3.72, 0.24 | 0.086 | -2.07 | -3.10, -1.04 | < 0.001 |
| **Note:** aβ: adjusted beta coefficient. aOR: adjusted odd ratio. CI: confidence interval; EWEs: Extreme weather events. Intervention (Season) effect calculated using generalized estimating equation linear/logistic regression model with an unstructured correlation matrix. Adjusted intervention (Season) effect, controlling covariates (gender, age, education, employment change, relationship status, motherhood), EWEs, and baseline outcome scores. | | | | | | | |
